# Harnessing GLP-1 Receptor Agonists for Obesity Treatment: Prospects and Obstacles on the Horizon

**DOI:** 10.1155/jobe/9919810

**Published:** 2025-11-24

**Authors:** Riad Mohammed Abdelrahman, Taha Hussein Musa, Ismail Adam Arbab, Mohsen Hussein Suliman, Eltieb Omer Ahmed, Asma Noureldaim Mohamed, Hassan Hussein Musa, Mohammed Jalal, Sahar Ibrahim Gasmallah

**Affiliations:** ^1^Faculty of Graduate Studies and Scientific Research, National Ribat University, Khartoum, Sudan; ^2^Faculty of Pharmacy, National Ribat University, Khartoum, Sudan; ^3^Pharmacy Program, School of Medical & Health Sciences, Libyan International University, Benghazi, Libya; ^4^School of Medicine & Biomedical Research Institute, Darfur University College, Nyala, Sudan; ^5^Department of Pharmacy Practice, Faculty of Pharmacy, International University of Africa, Khartoum, Sudan; ^6^Faculty of Medical Laboratory Sciences, University of Khartoum, Khartoum, Sudan; ^7^Faculty of Pharmacy, Karary University, Khartoum, Sudan; ^8^College of Public and Environmental Health, University of Bahri, Khartoum, Sudan

**Keywords:** GIP-1, GLP-1RAs, liraglutide, obesity, semaglutide, tirzepatide

## Abstract

**Background:**

Obesity has emerged as a pressing global health challenge, and therapies based on glucagon-like Peptide 1 receptor agonists (GLP-1RAs) have transformed its management. Currently, liraglutide, semaglutide, and tirzepatide are FDA-approved for obesity treatment, while other agents are used off-label. These drugs not only provide unprecedented efficacy and acceptable safety in weight reduction and glycemic control for patients with obesity and Type 2 diabetes but also hold promise in broader indications, including neurodegenerative disorders, fatty liver disease, dyslipidemia, atherosclerosis, and cardiovascular conditions.

**Methods:**

This narrative review examined the therapeutic applications of GLP-1RAs for obesity, emphasizing their efficacy, safety profile, challenges with patient adherence, and limitations. The review also explored emerging innovations such as ultralong-acting formulations, combination therapies, and the integration of digital health and artificial intelligence in advancing antiobesity drug development.

**Results:**

GLP-1RAs represent a paradigm shift in the treatment of obesity and metabolic diseases, with rapidly expanding indications and global uptake. Recent evidence highlights improvements in tolerability, global accessibility, and the potential of novel technologies to optimize patient outcomes. By 2025, GLP-1RAs are anticipated to receive FDA approval for new indications, such as chronic kidney disease, heart failure with preserved ejection fraction, and metabolic dysfunction–associated steatohepatitis. Novel agents including CagriSema and higher dose oral semaglutide are advancing through clinical trials, while pivotal trial results for orforglipron, mazdutide, retatrutide, and survodutide are anticipated to further expand the therapeutic landscape. At the same time, the arrival of generic liraglutide and evolving insurance coverage are reshaping access and affordability.

**Conclusion:**

The convergence of pharmacological innovation, digital health strategies, and equitable care initiatives is expected to revolutionize obesity therapeutics in the coming decade. Priorities for future research include sustaining long-term weight loss, establishing disease-modifying potential in nonmetabolic disorders, and addressing health equity concerns to ensure broader global benefit.

## 1. Introduction

Glucagon-like Peptide 1 receptor agonists (GLP-1RAs) have become a crucial category of treatments for Type 2 diabetes mellitus (T2DM) and obesity [1].

The established and emerging roles of GLP-1RAs critically examine their efficacy, safety, financial burden, and disparities in access, limitations, and prospects. While GLP-1RAs were initially developed for glycemic control in T2DM, accumulating evidence supports their utility in managing obesity independent of diabetes. Agents such as liraglutide, tirzepatide, and semaglutide have demonstrated clinically meaningful weight loss, prompting regulatory approvals specifically for obesity treatment [2].

### 1.1. Obesity: Overview and Impact

Obesity has grown into a major global health issue, driven by an imbalance between energy intake and expenditure, leading to excess fat storage. It is linked to multiple comorbidities like diabetes, arthritis, angina, and hypertension [3, 4].

Definitions and classifications vary, but WHO and CDC guidelines define overweight as a BMI ≥ 25 and obesity as a BMI ≥ 30. Traditional BMI measures are limited and carry stigma, prompting efforts like the American Association of Clinical Endocrinologists' (AACE) 2017 redefinition of obesity as “Adiposity-Based Chronic Disease” (ABCD) to focus on health impacts rather than weight alone [5, 6].

Globally, the prevalence of obesity has increased, with rising impacts in developing countries. As of 2022, 43% of adults were overweight, and 16% were obese. Managing obesity requires realistic goals, lifelong commitment, and a multidisciplinary approach, including primary, secondary, and tertiary prevention strategies. Lifestyle interventions often have limited success due to complex biological factors promoting weight regain [6–10].

### 1.2. Pharmacological Background

Pharmacologic treatments have evolved from toxic early options like dinitrophenol and amphetamines to modern drugs including orlistat, naltrexone, bupropion, phentermine, topiramate, and GLP-1RAs [11].

GLP-1, a hormone discovered in 1984, plays a crucial role in glucose regulation and has become a promising target for both diabetes and obesity treatments.

**Figure 1:**
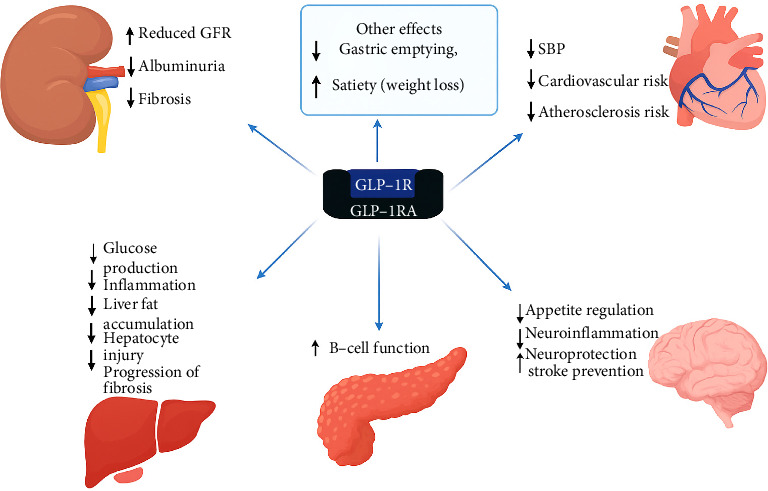
Diagrammatic representation of the diverse effects of GLP-1RAs across body organs and systems. *Source:* Adapted from B. P. Dave, Mol. Biol., 2024 [12].

Figure 1 illustrates the diverse physiological effects of GLP-1RAs across major organ systems, including the pancreas, liver, brain, cardiovascular system, kidneys, gastrointestinal tract, and adipose tissue.

The pharmacological profile of the GLP-1RAs is characterized by glucose-dependent insulin secretion, delayed gastric emptying, appetite suppression, and weight reduction, and has led to widespread clinical adoption [1].

GLP-1RAs vary in pharmacokinetics and dosing schedules (daily or weekly). Although generally safe and effective for modest weight loss, off-label use for weight loss has created access challenges for T2DM patients [3, 13–15].

### 1.3. Current Food and Drug Administration (FDA)–Approved and Investigational GLP-1RAs

Exenatide, the first FDA-approved GLP-1RA (2005), showed potential for significant weight loss, though more evidence is needed for use in treating uncomplicated obesity [16–19]. On April 15, 2014, the FDA approved albiglutide as adjunct therapy for glycemic control in T2DM; it was withdrawn in August 2017 due to low demand, not safety concerns [20, 21].

In 2014, liraglutide was approved for chronic weight management in patients aged ≥ 12 years meeting specific BMI thresholds [19, 22].

Dulaglutide was approved in the same year for T2DM but not for weight loss, and although it leads to modest weight reduction, this effect is significantly less than that achieved with semaglutide or liraglutide in obesity-focused trials [23–26].

Lixisenatide, approved in 2016, enhances glycemic control in T2DM and has shown tolerable weight reduction but lacks FDA approval for weight management. Clinical studies such as the GetGoal trial program demonstrated that while it improved glycemic control, its effect on body weight was small (typically ∼2–3 kg reduction) and not sufficient to warrant an obesity indication. As a result, regulatory approval was restricted to Type 2 diabetes management rather than obesity [27–29].

Beinaglutide was approved in China in 2016 for T2DM and also reduced BMI in overweight patients [13].

Semaglutide was approved in 2021 for chronic weight management in adults with obesity or overweight plus at least one weight-related condition; oral and injectable forms are FDA-approved for T2DM, but only the injectable form is approved for obesity. Semaglutide has received FDA approval for cardiovascular event risk reduction in 2020 and, most recently, kidney disease risk reduction in 2025 [14, 15, 30–32].

Tirzepatide, a dual agonist of GIP and GLP-1 receptors, was approved for T2DM in 2022 and obesity/overweight in 2023 [33]. The investigational triple agonist retatrutide achieved up to 17.5% mean weight reduction at 24 weeks [34].

In a pivotal phase 2 randomized trial published in the New England Journal of Medicine, orforglipron, a once-daily oral nonpeptide GLP-1RAs, demonstrated substantial and sustained weight loss among adults with obesity, with efficacy comparable to injectable agents and an acceptable safety profile [35].

A robust pipeline of entero-pancreatic hormone-based therapies is currently in development, with several dual and triple agonists expected to emerge for obesity in the coming years. These therapies are expected to offer greater efficacy and potentially target multiple metabolic pathways, providing improved outcomes for weight loss and related comorbidities like mazdutide, retatrutide, and survodutide [36, 37].

Retatrutide is a novel investigational agent that simultaneously activates GLP-1, glucose-dependent insulinotropic polypeptide (GIP), and glucagon receptors. This unique mechanism is designed to combine the incretin effects of GLP-1 and GIP with the energy-expenditure benefits of glucagon receptor activation, thereby potentially achieving greater weight reduction and metabolic improvements compared with single- or dual-agonist therapies. We have highlighted this in the updated section on emerging therapies [38].

To date, only liraglutide, semaglutide, and tirzepatide are FDA-approved for obesity or overweight [33]. A summary of FDA-approved GLP-1RAs is depicted in Table 1.

### 1.4. Long-Term Safety

GLP-1RAs are generally safe and effective in modestly reducing weight [39–42]. Common gastrointestinal side effects include nausea, vomiting, diarrhea, reflux, and constipation; rarer risks include pancreatitis. The FDA has issued boxed warnings regarding potential thyroid tumor risk, although human evidence remains inconclusive [43–47]. Beyond metabolic disorders, preliminary studies suggest potential benefits in cardiovascular disease prevention, neurodegenerative disorders, and nonalcoholic steatohepatitis (NASH), though further validation is needed [48].

Several observational studies have examined the potential association between GLP-1RAs and suicidality, with mixed findings regarding suicidal ideation, self-harm, and suicide risk [49]. Proposed biological mechanisms include hyperactivation of the hypothalamic–pituitary–adrenal (HPA) axis and the psychological impact of rapid weight loss, which may increase vulnerability to suicidality [50]. However, a preliminary review by the U.S. FDA found no evidence of a causal link between GLP-1RAs and suicidal thoughts or behaviors, while continuing safety monitoring [33].

## 2. Methodology

This narrative review was conducted to provide a comprehensive overview of the therapeutic applications of GLP-1RAs in obesity management. The objectives of this review were to evaluate the efficacy and safety of GLP-1RAs, examine patient adherence and clinical challenges, highlight emerging innovations such as ultralong-acting formulations and combination therapies, and explore the potential integration of digital health and artificial intelligence (AI) in obesity care.

### 2.1. Literature Search and Inclusion Criteria

This narrative review was based on a targeted search of PubMed, Scopus, and Web of Science, as well as official regulatory agencies, manufacturers of GLP-1RAs, and specialized entities providing economic estimates, to identify relevant publications on GLP-1RAs and obesity. Only English-language articles were considered. We included original research studies, clinical trials, review articles, and relevant commentaries that addressed the efficacy, safety, therapeutic potential, or emerging innovations of GLP-1RAs. The selected literature was synthesized to provide a comprehensive overview of current trends, clinical outcomes, limitations, and future directions in the use of GLP-1RAs for obesity treatment.

### 2.2. Long-Term Use Data and Adherence

In terms of adherence, semaglutide and tirzepatide have been associated with high adherence rates despite their respective once-weekly dosing. In contrast, liraglutide, with its daily administration, had a lower adherence profile [47, 51].

There is considerable individual variability in tolerance and response to GLP-1RAs, thereby influencing how a person reacts to these drugs. The needed dose and the duration of effect all depend on the patient's biology, physiology, and lifestyle. Recent studies among obese individuals without diabetes found poor 1-year persistence and adherence. Reasons for low rates of staying on GLP-1RAs therapy may include shortages as well as side effects [52–54].

The GLP-1RAs provide superior options for sustained obesity therapy. However, information regarding the effects of prolonged usage is under investigation. Numerous randomized controlled trials demonstrate that all FDA-approved anti-obesity drugs are both safe and effective for prolonged usage when prescribed judiciously to the suitable patient demographic, informed by a personalized health risk evaluation [51].

To achieve an effective weight loss regimen, physicians should prescribe anti-obesity drugs in conjunction with counseling, lifestyle modifications, consistent monitoring, and follow-up appointments. However, the reality is that it takes decades to accumulate pharmacological long-term safety data; clinical endocrinologists ought to be very well-versed in the most common GI-related side effects, including nausea, sometimes vomiting, reflux, constipation, and diarrhea, along with rarer risks, such as pancreatitis, and the FDA black box warning for medullary thyroid cancer [52].

The semaglutide treatment effect in people with obesity (STEP) clinical trial assessed the safety and efficacy of semaglutide; the STEP 5 trial represents the most extensive study on the use of semaglutide for weight management to date. All participants received a standardized lifestyle intervention, which included counseling on a reduced-calorie diet (≈500 kcal/day deficit) and increased physical activity (≥ 150 min/week) in addition to study drug or placebo. The study results showed that treatment with once-weekly subcutaneous semaglutide was associated with clinically impactful and sustained weight loss of 15.2% at Week 104 (95% CI, −15.3 to −9.8; *p* <  0.0001), along with improvements in weight-related cardiometabolic risk factors [53].

The satiety and clinical adiposity—liraglutide evidence (SCALE) evaluated once-daily 3.0 mg liraglutide for weight loss, metabolic health, and Type 2 diabetes prevention in individuals with overweight or obesity. Participants received lifestyle counseling with recommendations for a hypocaloric diet (≈500 kcal/day deficit) and increased physical activity, alongside trial medication. After 56 weeks, the study participants in the liraglutide group achieved an 8.4% weight loss compared to 2.8% in the placebo group (95% CI: −6.0 to −5.1; *p* < 0.001). Additionally, a total of 63.2% of the patients in the liraglutide group as compared with 27.1% in the placebo group lost at least 5% of their body weight (*p* < 0.001), and 33.1% and 10.6%, respectively, lost more than 10% of their body weight (*p* < 0.001) [54].

The SURMOUNT 4 is a trial to assess the effects of tirzepatide, a dual receptor agonist targeting GLP-1 and GIP, on long-term weight reduction over 2 years in those without diabetes. During the initial 36-week open-label treatment period, participants received background lifestyle intervention, including counseling on a healthy diet and physical activity, consistent with obesity management guidelines. Among patients completing the 36-week lead-in, tirzepatide produced sustained weight loss, with a mean reduction of 25.3% at 88 weeks versus 9.9% with placebo. From Weeks 36 to 88, weight change was –5.5% with tirzepatide compared to +14.0% with placebo (difference −19.4%; *p* < 0.001). Notably, 89.5% of tirzepatide recipients maintained at least 80% of their initial weight loss versus 16.6% with placebo (*p* < 0.001) [55].

### 2.3. Escalating Use of GLP-1RAs in Obesity Management

GLP-1 agonists, such as semaglutide, received significant attention in 2023 [56]. Since the FDA approved exenatide in 2005, GLP-1RA drugs have played a major role in diabetes treatment [57].

Over time, five FDA-approved GLP-1RA therapies for Type 2 diabetes, four of which are also approved for cardiovascular conditions. Although their potential for weight loss has long been recognized, FDA approval for obesity treatment has only occurred within the past decade [58].

The GLP-1RA market is on track for substantial expansion, as many companies prepare to introduce new injectable and oral options. An analysis of the clinical trial pipeline shows 39 GLP-1RA drugs in development across 34 companies, with only seven coming from the top 20 pharmaceutical firms. Notably, a significant number of these new entrants are based in China, suggesting a possible shift in market dynamics unless major partnerships or acquisitions take place [59].

Prescription rates for obesity medications are rising, driven by increased interest in GLP-1RA drugs. Starting in 2026, one to two GLP-1RA drug launches are expected annually, with the first oral version likely debuting by 2027. Both oral and injectable GLP-1RA treatments will continue to evolve, with approximately seven U.S. launches projected by 2030, factoring in development risks [60].

In the USA, GLP-1RAs showed a 700% increase over four years from 2019 to 2023 in the number of patients without diabetes starting treatment; about 60% were females [61].

A shortage of medicines containing GLP-1RAs has been affecting EU Member States since 2022. With the ongoing surge in demand for GLP-1RA drugs for weight loss, many individuals with Type 2 diabetes who need these medications to manage their disease have faced limited access [62].

Reports showed that a deficiency of GLP-1RA medications occurred in late 2022. The persistent deficiency of GLP-1RAs constitutes a significant public health issue that is improbable to be rectified in 2024, cautioned the European Medicines Agency. Consequently, the authorities have provided advice for the industry and healthcare practitioners to mitigate the adverse impacts of shortages on individuals with Type 2 diabetes and obesity [63].

A recent study indicated a consistent increase in prescriptions for obesity management medications in the United States, rising from 0.76 to 0.80 million between July 2017 and June 2018 and from 1.29 to 1.51 million between March 2023 and February 2024. The steady increase in prescriptions for FDA-approved obesity drugs led to an annual growth rate of 5.3%. Among the several obesity therapies, phentermine, semaglutide, and tirzepatide exhibited the greatest prescription rates, with approximately 0.74 million, 0.42 million, and 0.25 million monthly prescriptions, respectively, as of February 2024. The research additionally examined drugs not specifically sanctioned for obesity that include identical active components under several names to investigate potential off-label applications of GLP-1RAs [64].

### 2.4. Trends in the GLP-1RA Market

The study on the antiobesity medications shows an exponential growth in recent years. Prescription trends reflect increasing clinician and patient acceptance, partly driven by robust clinical trial data and heightened public awareness of obesity as a chronic disease. Semaglutide and tirzepatide (though technically a dual GIP/GLP-1 agonist) have experienced substantial demand, influencing global supply chains and pricing strategies [65].

**Figure 2:**
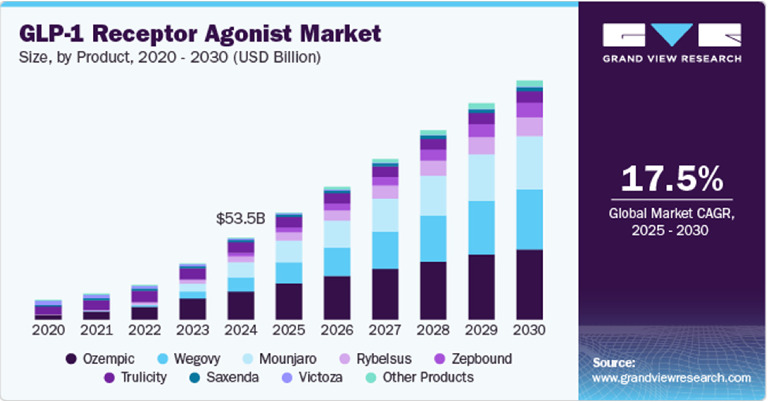
Projected global market growth of GLP-1RAs (2024–2030). *Source:*https://www.globaldata.com/store/report/glp-1r-agonists-in-type-2-diabetes-and-obesity-major-market-analysis/ on April 28, 2025.

The global market size for GLP-1RAs was valued at USD 53.46 billion in 2024, with North America accounting for 77% of the total revenue share. The market is projected to reach USD 70.08 billion in 2025 and USD 156.71 billion by 2030, expanding at a compound annual growth rate (CAGR) of 17.46% from 2025 to 2030 (Figure 2). This strong growth trajectory reflects the increasing adoption of GLP-1RAs in both Type 2 diabetes and obesity management.

**Figure 3:**
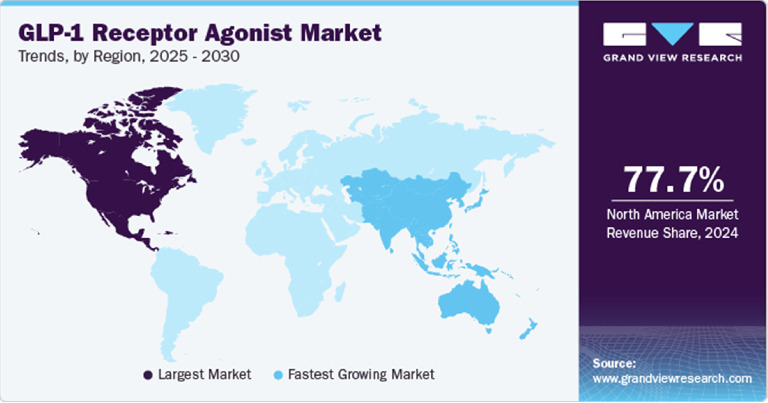
Regional distribution of GLP-1RAs revenues in 2024 (North America). *Source:*https://www.globaldata.com/store/report/glp-1r-agonists-in-type-2-diabetes-and-obesity-major-market-analysis/ on April 28, 2025.

Regional analysis highlights North America as the dominant market, maintaining over three-quarters of the global revenue share in 2024, driven by the high prevalence of diabetes and obesity, favorable reimbursement policies, and early adoption of innovative therapies (Figure 3). Countries with significant sales include the United States, Canada, Mexico, the United Kingdom, Germany, France, Italy, Spain, Denmark, Sweden, Norway, China, Japan, India, South Korea, Australia, Thailand, Brazil, Argentina, South Africa, the United Arab Emirates, Kuwait, and Saudi Arabia [66].

The introduction of new GLP-1 receptor agonists, a robust product pipeline for diabetes and obesity treatments, together with the high efficacy of these medications, is expected to drive market growth [67].

Despite this growth, access to GLP-1RAs remains uneven globally. High costs, limited insurance coverage, and regulatory differences restrict availability, particularly in low- and middle-income countries (LMICs). Supply chain limitations, cold-chain requirements, and healthcare provider familiarity further affect accessibility. Addressing these challenges will require strategies such as price negotiations, inclusion in essential medicines lists, and the integration of digital health tools to support adherence and real-time monitoring. Ensuring equitable access is essential not only for reducing individual obesity-related morbidity but also for mitigating broader societal and economic impacts.

### 2.5. Cost Impact and Equity of Access

The high cost of GLP-1RAs presents a significant barrier to widespread access, especially in LMICs. Disparities in healthcare coverage and reimbursement policies further exacerbate inequities in treatment availability. Economic evaluations and policy interventions will be crucial to ensure equitable access to these therapies, especially as they become more broadly indicated [68].

From 2018 to 2023, GLP-1RA prescriptions increased by 300%, owing to its efficacy, safety, and simplicity. Nonetheless, apprehensions persist regarding their elevated expense and the potential exacerbation of healthcare inequities [69, 70].

In the U.S. commercial market, the current estimated annual net prices for GLP-1RA drugs used to treat obesity range from $8000 to $9000. However, when purchased directly from manufacturers outside of insurance, these medications are offered at a lower price, around $499 per month or $5988 annually. This reflects a significant reduction from the $12,000 to $14,000 range cited in the Institute for Clinical and Economic Review's (ICER) 2022 evaluation. According to ICER's earlier cost-effectiveness modeling, today's net prices fall within what is generally considered reasonable long-term value in the U.S. healthcare system [68].

Despite this, fairness in such prices has been debatable. The critics opine that current prices aggravate inequities of access to proper treatment for obesity.

Even at reduced prices, pharmaceutical firms would still achieve gigantic profits far surpassing what normally results from other blockbuster medications, and lower prices would not damage innovation. They also point out that U.S. prices are significantly higher than in high-income European countries; for example, semaglutide costs roughly $8000 to $9000 per year in the United States, compared to approximately $2232 in Denmark, $1680 in Germany, and $1104 in the United Kingdom.

Furthermore, the same active ingredients are sold under different brand names at considerably lower prices when labeled for diabetes treatment rather than obesity. Semaglutide and tirzepatide in obesity formulations are priced 1.5 to 2.8 times higher than in their diabetes counterparts. Critics also highlight that brand-name GLP-1 manufacturers have secured extensive patent protection, averaging 19.5 patents per drug and 18.3 years of expected exclusivity, with over half of these patents focused on delivery devices rather than the drugs themselves [71].

Médecins Sans Frontières (MSF) estimates that GLP-1RAs diabetes medications cost $95 a month in Brazil, $115 in South Africa, $230 in Latvia, and $353 in the United States.

However, if generic manufacturers teamed up with branded companies to make them more widely available, prices could fall below $1 a month.

Companies' patents on medications remain intact, preventing the introduction of generic alternatives [72].

Also, high prices of GLP-1RAs due to their use in obesity management limit access to newer diabetes medicines in LMICs.

GLP-1RA drugs effective to treat obesity could account for 3%–4% of medical and Rx spend [73].

Consequently, numerous nations with public health systems confront challenging issues regarding the reimbursement of these medications and, if applicable, for particular demographics [74].

Addressing the cost and equity challenges of GLP-1RAs will require coordinated strategies, including policy interventions, generic competition, price negotiations, and innovative financing mechanisms, to ensure that these highly effective therapies are accessible to populations worldwide while maintaining incentives for continued pharmaceutical innovation.

Governments in LMICs should consider incorporating GLP-1RAs into national essential medicines lists and negotiating tiered pricing with manufacturers. Regional procurement mechanisms, such as pooled purchasing initiatives through the WHO or regional health organizations, could substantially lower costs. Strengthening local regulatory capacity and supporting technology transfer to enable generic or biosimilar production are additional avenues to enhance affordability. Public–private partnerships and outcome-based reimbursement models may further facilitate equitable access while ensuring sustainability in health budgets.

### 2.6. Ultralong-Acting GLP-1RAs and Combination Therapy

Developments in peptide engineering and drug delivery sciences have facilitated the development of ultra-long-acting GLP-1RAs, such as once-monthly injectables. These devices aim to enhance compliance through reduced injection frequency with preserved efficacy. As they progress through clinical pipelines, ultra-long-acting GLP-1RAs may redefine chronic obesity pharmacotherapy paradigms [60].

While the majority of patients appear content with the existing medications, a weekly injection can occasionally pose a strain for them. Consequently, prolonged-acting options permitting monthly administration may represent the forthcoming generation of obesity control pharmaceuticals, as stated by Global Data, a prominent data and analytics firm [75].

Global Data's recent report, “The State of the Biopharmaceutical Industry 2025 Edition,” emphasizes anti-obesity pharmaceuticals as the predominant trend expected to influence the business significantly in the forthcoming 12 months. The influx of investment in the obesity sector is propelling the advancement of potentially next-generation obesity pharmaceuticals, uncovering novel mechanisms of action, formulating oral alternatives to injectables, and aiming to alleviate patient burden by reducing administration frequency [76].

Amgen and Metsera are the two businesses that appear to be leading the development of a once-monthly dosage obesity medication.

Maridebart cafraglutide (known as MariTide) was a candidate to demonstrate significant efficacy in a once-monthly dosage regimen, with an average weight loss of 17% in patients at 52 weeks, with no plateau in weight loss. The mean percent change in body weight from the obesity cohort from baseline to 52 weeks ranged from −12.3% to −16.2% (95% CI, −15.0 to −9.7) to −16.2% (95% CI, −18.9 to −13.5). The obesity–diabetes cohort mean percent change in body weight ranged from −8.46% (95% CI, −11.0 to −5.7) to −12.3% (95% CI, −15.3 to −9.2) [77, 78].

Recently, the MET-097i candidate was originally developed for once-weekly administration, but its extended half-life also qualifies it for once-monthly dosing [62].

Combination pharmacotherapy with GLP-1RAs and other treatments (e.g., GIP agonists, SGLT2 inhibitors, or amylin analogs) is an exciting frontier for weight and metabolic benefit [79]. Combination therapies will likely prove additive or synergistic, preempting the plateau of monotherapy. Ongoing clinical trials will define the optimal combination and sequence.

Restricted studies integrating GLP-1 receptor agonists with alternative anti-obesity pharmaceuticals (e.g., GIP agonists, amylin analogs). Theoretical models indicate that GLP-1RAs and amylin analogs may function synergistically to promote weight loss [80].

### 2.7. AI in Antiobesity Drug Discovery

The application of machine learning (ML) and AI in drug discovery is accelerating the identification of novel targets and candidate compounds for obesity treatment [81]. Predictive modeling, virtual screening, and real-world data analysis are increasingly utilized to optimize lead compound selection and tailor treatment regimens, driving new advances in therapeutic innovation [82].

Digital solutions for care delivery also represent a promising avenue for enhancing access to overweight and obesity treatment, with the potential to scale rapidly to meet rising global demand. While many digital weight management tools, ranging from telehealth programs to personalized diet and exercise applications, remain insufficiently validated, their integration with evidence-based therapies has the potential to substantially improve outcomes [83, 84].

GLP-1RAs stand to benefit considerably from advances in AI and digital health. AI-driven platforms can accelerate the development of new GLP-1RA formulations and combination therapies, optimizing molecular design for greater efficacy and tolerability. Predictive modeling and real-world data analytics can help anticipate side effect profiles and refine patient selection, thereby improving both safety and cost-effectiveness. Furthermore, digital health tools can personalize dosing schedules, enhance adherence, and provide continuous monitoring of metabolic outcomes, ensuring that GLP-1RAs are used to their full therapeutic potential.

Thus, while AI and digital technologies are often discussed broadly in relation to obesity care, their greatest near-term impact may be in refining, optimizing, and extending the usefulness of GLP-1RAs, strengthening their role as cornerstone agents in modern obesity pharmacotherapy [85, 86].

## 3. Conclusion and Future Perspectives

GLP-1RAs represent a paradigm shift in obesity and metabolic disease treatment with expanding indications and growing global use. As the field further evolves, there should be an effort to improve tolerability, expand accessibility, and integrate novel technologies to optimize patient care. Long-term effectiveness, sustainability of weight loss, and disease-modifying capacity in nonmetabolic disease must also be prioritized in future research. The convergence of pharmacological innovation, digital health initiatives, and health equity initiatives provides the promise of revolutionizing obesity therapeutic landscapes over the coming decade.

In 2025, GLP-1RAs may gain FDA approval for new uses, including chronic kidney disease, certain types of heart failure, and liver disease. New injectable GLP-1s are advancing through clinical trials, with CagriSema (cagrilintide/semaglutide) among the most advanced, showing greater weight loss than semaglutide and expected to seek approval in late 2025. Oral GLP-1 mimetics are also emerging; higher dose semaglutide may soon be approved for obesity, while pivotal trial results for orforglipron, mazdutide, retatrutide, and survodutide are anticipated to further expand the therapeutic landscape. Insurance coverage and reimbursement policies for GLP-1RA medications may evolve in 2025 as payers respond to new evidence and indications. Generic liraglutide became available in 2024, though generics for newer GLP-1RAs such as semaglutide are unlikely before 2030. Beyond diabetes and obesity, GLP-1RA therapies have already received approvals for obstructive sleep apnea and chronic kidney disease, with further potential applications in heart failure with preserved ejection fraction (HFpEF) and metabolic dysfunction-associated steatohepatitis (MASH).

Looking forward, future research should focus on long-term safety, durability of weight loss, and real-world effectiveness of GLP-1RAs across diverse populations, including those in LMICs. Comparative effectiveness studies, pharmacovigilance programs, and registries will be critical to understanding rare adverse effects and long-term cardiometabolic outcomes. Moreover, investigations into the impact of treatment discontinuation, optimal sequencing with other therapies, and potential disease-modifying effects in cardiovascular, hepatic, and neurodegenerative disorders remain priority areas. Equally, policy-driven studies are needed to evaluate cost-containment models, equitable access strategies, and the public health implications of widespread GLP-1RA adoption. Such multidisciplinary efforts will ensure that the clinical promise of GLP-1RAs translates into sustainable global health gains.

## Figures and Tables

**Table 1:** FDA-approved GLP-1 RAs.

Trade name	Generic name	Population (indication)	Approval year	Key side effects
Byetta	Exenatide	Type 2 diabetes	2005	Nausea, vomiting, diarrhea, injection-site reactions, pancreatitis, hypoglycemia (with sulfonylureas/insulin)
Victoza	Liraglutide	Type 2 diabetes	2010	Nausea, diarrhea, vomiting, headache, pancreatitis, gallbladder disease, thyroid C-cell tumors (boxed warning)
Trulicity	Dulaglutide	Type 2 diabetes	2014	Nausea, vomiting, diarrhea, abdominal pain, decreased appetite, pancreatitis, injection-site reactions
Saxenda	Liraglutide	Obesity/overweight	2014	Nausea, vomiting, diarrhea, constipation, pancreatitis, gallbladder disease, thyroid C-cell tumors (boxed warning)
Adlyxin	Lixisenatide	Type 2 diabetes	2016	Nausea, vomiting, headache, dizziness, hypoglycemia (with insulin/sulfonylureas), pancreatitis
Xultophy	Liraglutide + insulin Degludec	Type 2 diabetes	2016	Hypoglycemia, weight gain, nausea, diarrhea, injection-site reactions, pancreatitis, thyroid C-cell tumors
Soliqua	Lixisenatide + insulin glargine	Type 2 diabetes	2016	Hypoglycemia, nausea, diarrhea, headache, weight gain, pancreatitis
Bydureon BCise	Exenatide	Type 2 diabetes	2017	Nausea, diarrhea, injection-site nodules/reactions, pancreatitis, hypoglycemia (with insulin/sulfonylureas)
Ozempic	Semaglutide	Type 2 diabetes	2017	Nausea, vomiting, diarrhea, constipation, abdominal pain, gallbladder disease, thyroid C-cell tumors (boxed warning), rare pancreatitis
Rybelsus	Semaglutide (oral)	Type 2 diabetes	2019	Nausea, diarrhea, abdominal pain, decreased appetite, vomiting, rare pancreatitis, gallbladder disease
Wegovy	Semaglutide	Obesity/overweight	2021	Nausea, vomiting, diarrhea, constipation, abdominal pain, headache, gallbladder disease, thyroid C-cell tumors (boxed warning), rare pancreatitis
Mounjaro	Tirzepatide	Type 2 diabetes	2022	Nausea, diarrhea, vomiting, decreased appetite, constipation, abdominal pain, gallbladder disease, possible thyroid C-cell tumors, pancreatitis
Zepbound	Tirzepatide	Obesity/overweight	2023	Nausea, vomiting, diarrhea, constipation, abdominal pain, gallbladder disease, pancreatitis, possible thyroid C-cell tumors

*Note:* Source: https://www.fda.gov/drugs/drug-safety-and-availability/update-fdas-ongoing-evaluation-reports-suicidal-thoughts-or-actions-patients-taking-certain-type.

## Data Availability

The data supporting the findings of this study are available on the websites mentioned in the References section.
